# Impact of livestock-associated MRSA in a hospital setting

**DOI:** 10.1186/s13756-015-0053-8

**Published:** 2015-04-17

**Authors:** Nienke van de Sande-Bruinsma, Maurine A Leverstein van Hall, Maria Janssen, Nynke Nagtzaam, Sander Leenders, Sabine C de Greeff, Peter M Schneeberger

**Affiliations:** Center for Infectious Disease Control, Epidemiology and Surveillance, National Institute for Public Health and the Environment (RIVM), Bilthoven, The Netherlands; Department of Medical Microbiology and Infection Control, Bronovo Hospital, The Hague, The Netherlands; Department of Medical Microbiology and Infection Control, Jeroen Bosch Hospital, PO Box 90153, ‘s-Hertogenbosch, 5200 ME The Netherlands

**Keywords:** MRSA, Infection control, Carriage

## Abstract

**Objectives:**

The Netherlands is known for a stringent search and destroy policy to prevent spread of MRSA. In the hospital setting, livestock-associated MRSA (LA-MRSA) is frequently found in patients coming from the high density farming area in the south of the Netherlands. The aim of the study was to determine the contribution of LA-MRSA in the epidemiology of MRSA in cases found following the Dutch search and destroy policy.

**Patients and methods:**

From two hospitals serving a population of 550,000 persons all data on MRSA cultures and subsequent control measures from 2008 and 2009 were retrospectively collected and analyzed.

**Results:**

A total of 3856 potential index patients were screened for MRSA, 373 (9.7%) were found to be positive, 292 ( 78%) LA-MRSA and 81 (22%) non-LA-MRSA respectively. No secondary cases were found among contact research in persons exposed to LA-MRSA (0/416), whereas similar contact research for non-LA-MRSA resulted in 83 (2.5%) secondary cases. LA-MRSA were rarely found to cause infections.

**Conclusions:**

LA-MRSA is more prevalent than non-LA-MRSA in Dutch Hospitals in the South of the Netherlands. However, retrospectively studied cases show that the transmission rate for LA-MRSA was much lower than for non-LA-MRSA. This suggest that infection control practices for LA-MRSA may possibly be less stringent than for non-LA-MRSA.

## Introduction

The Netherlands is known for a stringent search and destroy policy to prevent spread of MRSA in care facilities. According to the Dutch MRSA guideline for hospitals (www.wip.nl), patients at risk for being a carrier are screened for MRSA at admission. Defined risk groups are: known MRSA carriers, patients hospitalised abroad, or contacts of known MRSA carriers. Since june 2006, persons in close contact with pigs and veal before hospital admission have been included in the guideline, assuming equal clinical significance between LA-MRSA and healthcare-associated MRSA (HA-MRSA). When being admitted, asymptomatic MRSA carriers are treated in isolation to prevent transmission. The percentage of LA-MRSA among all isolates sent in to the national MRSA surveillance in the Netherlands is about 40% annualy [1]. Especially in the South of the Netherlands where the highest concentrations of livestock animal farming can be found, a high prevalence of LA-MRSA is assumed to lead to considerable increased workload and infection control precautions in hospitals. Still, in contrast to non-LA-MRSA, severe infections due to LA-MRSA seem to be rare [2] and the justification of these precaution efforts can be questioned.

The aim of this epidemiological study was to determine the prevalence of LA-MRSA and the contribution of LA-MRSA to the total epidemiology of MRSA patients screened conform the search and destroy policy in a high density farming area in the South of the Netherlands. Furthermore, we evaluated the relative clinical relevance and risk of transmission of LA-MRSA by comparing the transmission rate and infection rate of LA-MRSA and non-LA-MRSA.

## Materials and methods

### Study design

From the laboratory information system of the regional laboratory in Den Bosch located in a high density farming area in the South of the Netherlands (serving 2 hospitals, general practitioners and nursing homes and a population of about 550.000 persons), the test results of all screening cultures performed per patient for MRSA detection from 2008 and 2009 were collected.

In case of a MRSA positive result the following information was obtained: i) indication for MRSA screening i.e. according to the risk group categories of the MRSA guideline (www.wip.nl), ii) the institution where the culture was taken ((general practice, nursing home, outpatient department, first aid department, hospital), iii) the spa typing results from the national MRSA surveillance discriminating LA-MRSA (ST398) and non-LA-MRSA. If the MRSA was isolated from a clinical sample the medical records from the hospital information system were retrieved.

The clinical relevance of the culture was based on the material (blood, wound, urine) the isolate was cultured from. A MRSA carrier was defined as a patient being colonized and/or infected (superficial or deep infection) with MRSA. In case of transmission subsequent secondary contact tracing was attributed to the index case.

An index case was a patient that was found MRSA positive and resulted in contact research. Secondary cases were patients that were found MRSA positive during contact research of an index patient.

### Statistical methods

Characteristics of patients and isolates were analysed. Differences in percentages were tested with a Chi-square test or Fisher’s exact test, if appropriate.

## Results

### Detection of MRSA

**Figure 1:**
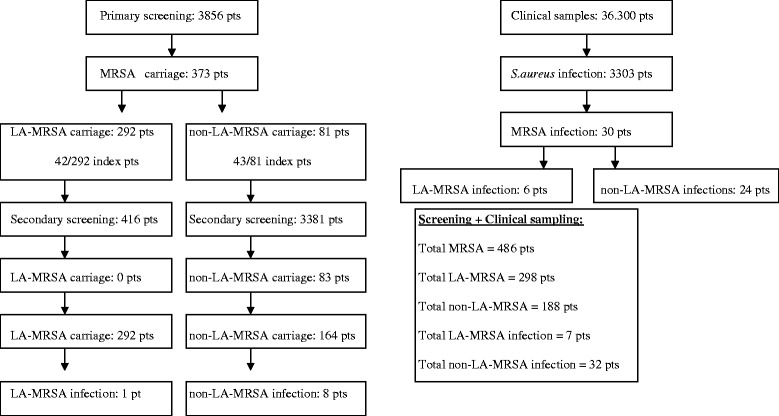
Study design 2008–2009.

In total 10,233 primary screening cultures from 3856 unique patients for MRSA were taken in the period 2008–2009. For 25% of the 3856 screened patients the indication of screening was available. From this group 26% of the patients were screened as they reported professional contact with pigs and or veal calves and 64% were screened for risk carrying non-LA-MRSA (i.e. contact with a non-LA-MRSA positive patient or recent admission in a high risk hospital) In total 373 (9.7%) of the 3856 patients were found to be colonized with MRSA through primary screening Of these, 292 (78%) were LA-MRSA and 81 (22%) were non-LA-MRSA (Figure 1).

Forty-two patients with LA-MRSA and 43 patients with non-LA-MRSA carriage resulted in contact research of 416 and 3381 patients and personnel respectively (secondary screening). No secondary cases were found among the contact research conducted for LA-MRSA patients (0/416), whereas the contact research of the non-LA-MRSA patients resulted in 83 (2.5%) secondary non-LA-MRSA cases (Figure 1).

Thirty patients were unexpectedly detected as MRSA carriers, when clinical cultures were taken. These infections were most likely community acquired, as none could be linked to a known index case. Six patients had an infection with a LA-MRSA strains (25%).

Overall, via primary (373) and secondary screening (83) and regular clinical culture (30), 486 patients were found to be MRSA carrier in 2008 and 2009 together (Figure 1).

### Indication of screening

**Figure 2:**
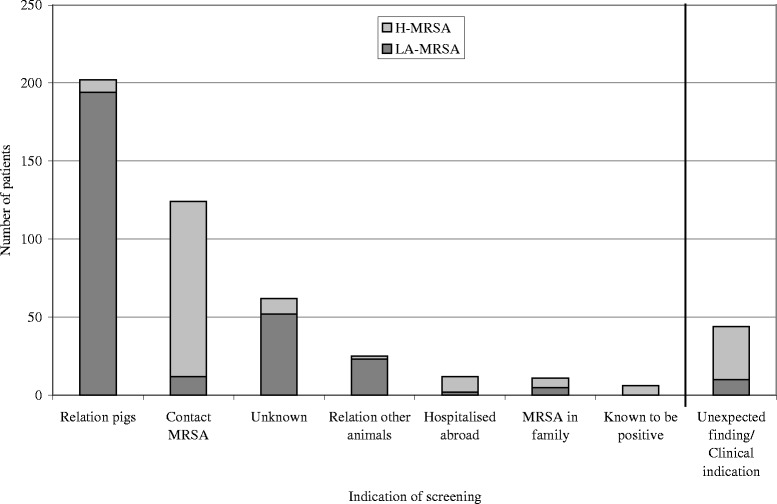
Indication of screening of the MRSA positive patients.

In Figure 2 the indication of screening for the MRSA positive patients is displayed, making a distinction between LA-MRSA (n = 292) and non-LA-MRSA (n = 81) patients. The patients with MRSA detected through secondary screening (n = 83) fall within the ‘contact MRSA’ column of non-LA-MRSA. A large diversity in the characteristics of the patients carrying LA-MRSA versus non-LA-MRSA was found (Table 1).

**Table 1:** **Patient characteristics of the patients colonized with LA-MRSA and non-LA-MRSA**

**Patient characteristics**	**LA-MRSA (n = 298)**	**non-LA-MRSA (n = 188)**
Male*	214 (72%)	84 (45%)
<= 15 years	21 (7%)	16 (9%)
>15 - < 65 years*	255 (86%)	99 (53%)
> = 65 years*	22 (7%)	73 (39%)
Colonized	291 (98%)	154 (82%)
Superficial infection*	3 (1%)	8 (5%)
Deep infection*	4 (1.3%)	24 (13%)
GP*	120 (40%)	47 (25%)
Nursing resident*	4 (1.3%)	82 (44%)
First aid*	41 (14%)	8 (4%)
Outpatient department*	64 (21%)	13 (7%)
Hospitalized*	20 (7%)	27 (14%)
Outpatient department or Hospitalized*	35 (12%)	5 (3%)

*p < =0.05.

### MRSA infections

During the study period, 39 patients had a MRSA infection (Figure 1). Seven of these infections were caused by LA-MRSA (Table 2). Only the first case, depicted in Table 2, was screened at forehand due to contact with pigs, and was therefore a known carrier. The 6 other LA-MRSA infections were unexpected findings and could not directly be linked to close contact with pigs. The spa types of the LA-MRSA isolates causing clinical infections were mainly dispersed among the most frequent spa types (t108, t011, t11) detected overall. In the 2 year study period no bacteraemia caused by a LA-MRSA was found and none of the patients died because of the LA-MRSA infection.

**Table 2:** **Medical history of the 7 patients with a LA-MRSA infection**

**Pt**	**Age/gender/Spa type**	**Type of infection**	**Treatment**	**Animal contact**	**Extra**
1	40/male/t011	External otitis	Surgical cleansing, no antibiotic treatment	Pig farmer	Cured
2	90/male/t567	Pleural empya	Surgical drainage, quinelones and rifampin	None	Post infectious sterile pleural effusion, surgically drained and secondarily infected through drain (colonized) with LA-MRSA
3	41/male/t011	Abscess finger	Incision and drainage no antibiotic treatment	Butcher	Abscess of trauma with knife, wound cured.
4	89/male/t108	Abcess tooth/jaw	Incision and drainage (2x) no antibiotic treatment	None	Cured
5	60/male/t108	Wound infection of lymphnode biopsie	No specific treatment	Horse farm	T cell lymphoma, wound infection cured
6	80/female/t011	Conjunctivitis	Topical antibiotics (fusidinic acid)	None	Conjunctivitis occurred during stay on ICU for gram negative urosepsis. Conjunctivitis cured
7	40/female/t011	Toe wound	Numerous antibiotics	None	Wound occurred after a visit to Turkey. Wound cured.

Of the 32 infections caused by non-LA-MRSA (Figure 1), 8 were known carriers through 113 screening and the remaining 24 non-LA-MRSA infections were unexpected findings. The majority were wound infections (50%) and abscesses (22%). Two non-LA-MRSA strains, that were both spa type t179, caused a bacteraemia. Both of these patients were not screened for MRSA. The spa types of the non-LA-MRSA isolates causing clinical infections were mainly dispersed among the most frequent spa types (t179, t002, t002, t311, t316, t008) detected overall. None of the patients died because of the non-LA-MRSA infection.

## Discussion

In this study, 59% of the detected MRSA carriers in 2008 and 2009, were colonized with LA-MRSA. The majority of patients for which we knew the indication of screening, were screened because of close contact with pigs and veal calves, contributing to 23% of the MRSA positive cultures, compared to 9% positive cultures for the other screening indications. These figures underscore the additional workload and costs related to implementation of the search and destroy measures caused by LA-MRSA in a high density farming area in the South of the Netherlands. Even though the majority of LA-MRSA was directly linked to close contact with pigs, LA-MRSA was also frequently found among patients that were screened for close contact with other livestock animals. Moreover, LA-MRSA was also found accidentally among patients without known contact with livestock, when they were screened for other reasons such as a contact with an known MRSA carrier (10%; 12/124, not related to outbreak investigation), MRSA carriage within the family (45%; 5/11) or hospitalization abroad (17%; 2/12) (Figure 2). Clearly, livestock and in particular pigs are an important reservoir of MRSA in the Netherlands [3-9], that should be closely monitored and possibilities to reduce transmission to humans should be investigated. However, our results in line with the findings of others that LA-MRSA is not solely attributed to close or direct contact with livestock, but in some cases may have dispersed in a community where the density of pig farms is high [10]. This adds to the increasing number of reports which found LA-MRSA isolated from patients in hospitals among individuals without known contact with livestock [11-15].

A major limitation of this study was the relatively large group of MRSA positive patients without a known indication for screening (n = 62, 13%), even after additional inquiry of the treating physician that requested the screening. Moreover, the majority (84%) of the patients with an unknown indication of screening carried LA-MRSA, suggesting that part of them failed to report contact with livestock. Questionnaires to register the risk group were submitted by the treating specialists. More than one risk factor was only sporadically indicated, suggesting a bias by under recording multiple risk factors.

During the 2 year study period an equal number of LA-MRSA and non-LA-MRSA index patients were indentified. In case of transmission, subsequent secondary contact tracing was attributed to the index case, and the sesecondary cases resulted in additional contacts which had to be screened. No secondary cases were found for LA-MRSA, whereas 84 secondary cases were found during the contact research for non-LA-MRSA. Consequently, for contact research of LA-MRSA on average 10 patients and personnel were screened, compared to 80 patients and personnel for each non-LA-MRSA contact. Individuals colonized with LA-MRSA are significantly less likely to transmit LA-MRSA to other individuals in the hospital setting compared to non-LA-MRSA [2,16]. We realize that secondary cases based on contact history and SPA typing my overestimate the actual number of secondary cases.

In a study performed in Dutch hospitals, LA-MRSA was found 4–6 times less transmissible than non-LA-MRSA [17,18]. After artificial inoculation LA-MRSA is capable to colonize the human nose for a prolonged period, however Van Cleef et al. showed that after short-term occupational exposure LA-MRSA was frequently present, but in most cases the strain was lost again after 24 hours [19,20]. This failure to actually colonize their host under certain circumstances may further explain the lower chance to transmit LA-MRSA.

Nevertheless, in contrast to our finding, outbreaks of LA-MRSA can occur in hospital and other health care settings [21,22]. However in nursing homes infection control practices may be less stringent than in hospitals [22].

It has been suggested that LA-MRSA may not cause as much disease as non-LA-MRSA strains [2]. Several studies have found that ST398 typically lacks many previously identified toxin genes [23-26], including the PVL gene, though pvl positive ST398 have been reported [4,25,27]. In the present study, 39 infections with MRSA were reported of which 7 were caused by LA-MRSA (18%). Only one of the 7 infected patients was screened at forehand due to close contact with pigs. The other six were detected through clinical sampling.

As mentioned earlier, only one of the 7 patients with a LA-MRSA infection had been screened at forehand. Among the 6 patients that were not screened at forehand, one turned out to be a butcher and one a horse farmer. The other 4 patients had no known contact with livestock. The butcher had cut his hand with a knife at his butchery and the horse farmer also had a wound infection. The Dutch Food Safety Agency has sampled various kinds of meat collected from retail trade. MRSA was isolated from 12% of the 2217 samples analyzed and the majority (85%) belonged to ST398 [8]. Several other studies from all over the world have also found MRSA in retail meat samples. Even though the chance of colonization with MRSA for professionals handling raw meat is low [28] it could be a plausible explanation in the Butcher’s case. The number of reports on MRSA colonization and infections in horses is increasing and varies between 0% and 4.7% on horse farms in Europe, Canada and North America [29-32]. In a recent study from the Netherlands, a suspected transmission of MRSA ST398 between a horse and a girl, resulting in a foot infection, was described [33].

A remarkable finding of the present study was that the majority of MRSA infections was found unexpectedly and was not among the screened patients with a known risk for MRSA. This supports the idea that MRSA in general is increasingly found outside the known reservoirs. In a study published by Lekkerkerk et al [34], the emergence of MRSA with no link to established Dutch risk factors for acquisition, hampering early detection and control, is described. They reported that from the Dutch national MRSA surveillance at least 24% of the 5565 MRSA isolates registered were not from a defined risk group and that studies on new sources and transmissions are urgently needed to control the spread of MRSA [34].

In conclusion, in the Netherlands, MRSA carriage and infection remains a rare finding, reflecting the success of the “Dutch” search and destroy policy. Although LA-MRSA appears to have a very limited impact on clinical disease and hospital transmission it does require extensive control measures which may effect the operation of control measures for non-LA-MRSA, especially in an area where the density of pig-farms and the prevalence of LA-MRSA is likely to be high.

If the transmission rate and infectious capabilities of LA-MRSA stay stable over time, rapid laboratory identification of LA-MRSA can facilitate infection control by rapidly distinguishing LA from non-LA-MRSA. Possibly, in hospitals control measures for LA-MRSA may be less stringent compared to non-LA-MRSA, since LA-MRSA does spread in the community but rarely seems to spread in hospitals.
